# Long-Term Clinical Responses of Neoadjuvant Dendritic Cell Infusions and Radiation in Soft Tissue Sarcoma

**DOI:** 10.1155/2015/614736

**Published:** 2015-12-31

**Authors:** Shailaja Raj, Marilyn M. Bui, Gregory Springett, Anthony Conley, Sergio Lavilla-Alonso, Xiuhua Zhao, Dungsa Chen, Randy Haysek, Ricardo Gonzalez, G. Douglas Letson, Steven Eric Finkelstein, Alberto A. Chiappori, Dmitry I. Gabrilovitch, Scott J. Antonia

**Affiliations:** ^1^H. Lee. Moffitt Cancer Center, Tampa, FL 33612, USA; ^2^MD Anderson Cancer Institute, Houston, TX 77030, USA; ^3^The Wistar Institute, Philadelphia, PA 19104, USA; ^4^21st Century Oncology, Scottsdale, AZ 85251, USA

## Abstract

*Purpose*. Patients with large >5 cm, high-grade resectable soft tissue sarcomas (STS) have the highest risk of distant metastases. Previously we have shown that dendritic cell (DC) based vaccines show consistent immune responses.* Methods*. This was a Phase I single institution study of neoadjuvant radiation with DC injections on 18 newly diagnosed high-risk STS patients. Neoadjuvant treatment consisted of 50 Gy of external beam radiation (EBRT), given in 25 fractions delivered five days/week, combined with four intratumoral injections of DCs followed by complete resection. The primary endpoint was to establish the immunological response to neoadjuvant therapy and obtain data on its clinical safety and outcomes.* Results*. There were no unexpected toxicities or serious adverse events. Twelve out of 18 (67%) patients were alive, of which an encouraging 11/18 (61%) were alive with no systemic recurrence over a period of 2–8 years. Favorable immunological responses correlated with clinical responses in some cases.* Conclusions*. This study provides clinical support to using dendritic cell injections along with radiation in sarcomas, which when used optimally in combination can help clinical outcomes in soft tissue sarcoma. Study registration number is NCT00365872.

## 1. Introduction

Although sarcomas are relatively rare neoplasms, more than 8,000 new cases of soft tissue sarcomas (STS) occur in the United States every year, making this disease a significant health problem affecting all age groups [1]. Despite appropriate curative therapies, patients with localized high-grade STS larger than 5 cm remain at significant risk of treatment failure and death from metastatic disease, with prognosis becoming ever more dismal with increased tumor size and higher grade [2–5].

Multimodality therapy, including radiation and at times systemic chemotherapy, in properly selected patients, can improve local control rate, decrease the morbidity associated with surgery and increase the duration of relapse-free survival [3–5]. Coindre et al. found that high-grade tumors had a metastasis-free survival rate of 44% [6] Earlier on, a meta-analysis of 14 trials comprising 1,568 patients, with localized resectable extremity and nonextremity primary STS [7], showed that the overall survival was not improved significantly by the use of chemotherapy. There was a trend towards improved survival with an absolute potential benefit of 4%, which translated to an overall survival improvement from 50% to 54%. An Italian Study showed that adjuvant chemotherapy in high-grade, over 5 cm, soft tissue sarcomas had a disease-free survival of 48 months with use of epidoxorubicin and ifosfamide [8]. This demonstrated a 41% relative reduction in the risk of disease relapse, translating to an absolute benefit of 27% from chemotherapy at two years and 13% at four years.

One potential approach to decrease the risk of recurrence in large, high-grade, soft tissue sarcomas is immunotherapy using vaccines. Vaccines can deliver tumor-associated immunogenic factors (antigens) that provide a Trojan horse effect [9]. The use of dendritic cells holds promise for sarcoma patients who are often resistant to chemotherapy. Intratumoral injection of dendritic cells is one of the methods that can utilize a large number of antigens from tumors without the need for their selection based on HLA typing or expression. Manipulation of the tumor microenvironment by ionizing radiation is also an important contributing factor in addition to the antitumor effects caused by the dendritic cells. Radiation therapy on its own can induce an immune response; for example, external beam radiotherapy can trigger signals that stimulate Toll-like receptor four on DCs [10, 11]. Radiation also has a direct effect on tumors by rendering them more susceptible to vaccine-mediated T-cell killing or releasing cytokines and inflammatory proteins. Finally, it can alter the tumor microenvironment to promote greater infiltration of immune effector cells [12–14].

We tested the hypothesis that the combination of dendritic cell vaccine and radiation would augment immune and antitumor responses in a preclinical murine model. This was done using dendritic cell administration in two well-characterized mouse tumor models, MethA sarcoma and C3 tumor, which carry defined MHC class I-restricted tumor-specific antigens. The study first found that the number of labeled dendritic cells trafficking to the tumors dramatically increased if the tumor was irradiated before subcutaneous administration of the dendritic cells. Next, it showed that when dendritic cell vaccination and radiation were combined in the MethA sarcoma mouse group, by day 14, 10 of 12 mice rejected their tumors completely. In the C3 tumor model, the combination resulted in significantly reduced tumor growth, with 4 of 10 mice rejecting their tumors and the remainder showing slowed tumor growth [15]. Other preclinical studies have also shown that combining local tumor irradiation with intratumoral DC administration resulted in potent antitumor immune responses that translated into an antitumor effect [16]. Immune escape has been demonstrated both in GIST [17] and Ewing's sarcoma [18, 19].

We then sought to translate these preclinical findings into a Phase I trial, to focus on the feasibility, safety, and immunological responses of dendritic cell vaccine with radiation followed by surgery. The primary endpoint was immunological responses that have been previously reported. Here, our primary aim is to provide an update of the long-term clinical results of this neoadjuvant trial that treated newly diagnosed, high-risk soft tissue sarcoma patients with a combination of DC injections and radiation. Our second aim is to compare immune responses to the clinical responses to DC injections with radiation combined in the treatment of high-grade soft tissue sarcomas over 5 cm in size.

## 2. Methods

### 2.1. Patient Selection

Patients over age 18 with histologically confirmed > 5 cm intermediate or high-grade STS of the extremity/trunk/chest wall were eligible for enrollment. Patients were required to have a World Health Organization (WHO) performance status of 0 or 1, no exposure to steroids in the first four weeks, adequate organ function, no coagulation disorders, and no contraindication to resection. A radiation oncologist had to confirm that each patient had a 2-3 cm strip of skin that could be spared from radiation before the start of treatment. Patients with severe allergic reactions, GIST, retroperitoneal sarcomas, metastatic disease and chronic intercurrent illness, prior radiation therapy, severe immunosuppressive disease or HIV, or previous autoimmune disease were all excluded. All patients were clinically confirmed Stage III, with a significant risk of progressing to distant metastases.

### 2.2. Preparation and Administration of DCs

Expanded autologous DC can be routinely obtained from mononuclear cell fraction progenitors obtained from individuals using leukapheresis, followed by* ex vivo* expansion with standardized protocols [20]. DCs collected in this manner are HLA-matched (autologous) and are competent to take up tumor-derived apoptotic bodies and present tumor antigens to lymphocytes. DCs (10^7^ cells) with a phenotype lineage (CD3, CD14, CD19, CD20, and CD56) negative and HLA-DR positive were injected in a total volume of 1 mL.

### 2.3. Evaluation of DC Migration

DCs labeled with ^111^In and 5 × 10^6^ cells were injected intratumorally before surgery. DC labeling and imaging were published previously [12].

### 2.4. Study Design and Treatment

We had enrolled eighteen patients at Moffitt Cancer Center, who were analyzed for clinical and immunological outcomes. They were required to provide written informed consent to a University of South Florida Institutional Review Board that previously approved the protocol.

Patients were treated with external beam radiation (EBRT) 50 Gy in 25 equal fractions, delivered five days per week (Monday–Friday), combined with 10^7^ dendritic cells in a total volume of 1 mL, and injected intratumorally, three times on the second, third, and fourth Fridays during the radiation. One additional DC injection was given several days before surgery. Surgical resection of tumors occurred 3–6 weeks after completion of radiation.

### 2.5. Coordinating Radiation Therapy and Dendritic Cell Injections

To maximize the immunological response, the radiation therapy was coordinated to ensure that adequate apoptosis (forming apoptotic bodies for uptake into DCs with subsequent tumor antigen presentation) was ongoing within the soft tissue sarcoma at the time of intratumoral dendritic cell injection. No studies of serial sarcoma tumor biopsies after external-beam radiation to establish the time course of apoptosis have been published to our knowledge. In our protocol, dendritic cell injections were given after 18, 27, 36, and 50.40 Gy of radiation. Dendritic cells were injected on Friday morning after completing the radiation fraction for that day as this ensured that there was a period of 48 hours before the next radiation treatment, thus minimizing the risk of inactivation of DCs.

### 2.6. Evaluation of Immune Responses

Survivin is an antiapoptotic protein that preferentially blocks mitochondrial-dependent apoptosis by targeting caspase 9 [20, 21]. Overexpression of this protein has been documented in many tumors including soft tissue sarcomas. Survivin-specific immune responses in healthy individuals and prostate-cancer patients have been measured by our group previously [20]. We planned to evaluate the survivin-specific responses in this trial. For this, peripheral blood mononuclear cells (PBMC), tumor cells harvested through core biopsies, and tumor cell lysates (TCL) prepared by repeated snap freeze-thawing cycles were stored in liquid nitrogen, as described [22]. Two sources of tumor-associated antigens (TAAs) were used to evaluate comparatively the immune response in patients: whole tumor cell lysate (TCL) and survivin. T-cell responses to TCL were assessed using IFN-*γ* ELISPOT [23] and proliferation assays. To evaluate T-cell response to survivin, DCs were infected with adenovirus-survivin (Ad-surv), as described previously, to serve as stimulator cells [23].

The immune response of an individual patient was considered to be positive if 2 criteria were met: first, if at any time point the response in the IFN-*γ* ELISPOT assay was higher than 30 spots per 2 × 10^5^ cells or if the 3[H]-thymidine counts in the proliferation assay were greater than 3000 CPM; second, when the response in the IFN-*γ* ELISPOT assay or proliferation assay was greater than two standard deviations than the control lysate at the same time point and two standard deviations greater than the response at baseline before the start of treatment.

### 2.7. Statistical Analysis

We used GraphPad Prism Software 6 for statistical analysis. Univariate Chi-square tests were used to test differences in demographics or clinical characteristics; Kaplan-Meier and log-rank tests were employed for determining survival outcomes.

## 3. Results

### 3.1. Study Characteristics

From May 2007 to January 2009, we enrolled a total of 18 patients (ClinicalTrials.gov identifier NCT00365872). The demographics and patient characteristics are described in Table 1. The median age group was 20–80 years with 77% male and 23% female.

### 3.2. Safety

The overall toxicities are summarized in Table 2. With the four doses of dendritic cell injections, we recorded injection site reactions, fatigue, nausea, pain, and constipation. There were no grades 4 or 5 toxicities and hepatic or renal events and no treatment-related deaths observed. Additionally no unexpected serious adverse reactions including autoimmune events were reported. Overall the intratumoral method of dendritic cell injections was well tolerated and together with radiation was a feasible mode of treatment.

### 3.3. Clinical Outcomes

Clinical follow-up was available for all individuals for a minimum period of twenty-four months after primary DC immunization and those who were alive, to over eight years. Amongst the eighteen vaccinated patients twelve (67%) remain alive with no disease recurrence. Eight patients (45%) are alive between 6 and nine years and three over four years, while one patient was lost to follow-up after three years. Interestingly one patient, who progressed with lung metastases in 2 years, remains alive for eight years, being treated on other protocols. The remaining six (33%) died of progressive metastatic disease to the lung between seven months and three years after treatment.

The disease-free survival at two years is 83% and at four years is 67%. The log-rank tests for trend showed a significant trend at *p* = 0.04, whereas, by Gehan-Wilcoxon test, the results were not statistically significant. The median survival was 57 months.

Of the 18 eligible patients, 6 had progressed, and 5 have died. Of the deaths, 5/30 (17%) were confirmed deaths from progressive metastatic disease to the lung. Of the 12 patients, last known to be alive as of April 28, 2015, the median follow-up is 4.4 years (range 0.7–8.7 years).

**Figure 1:**
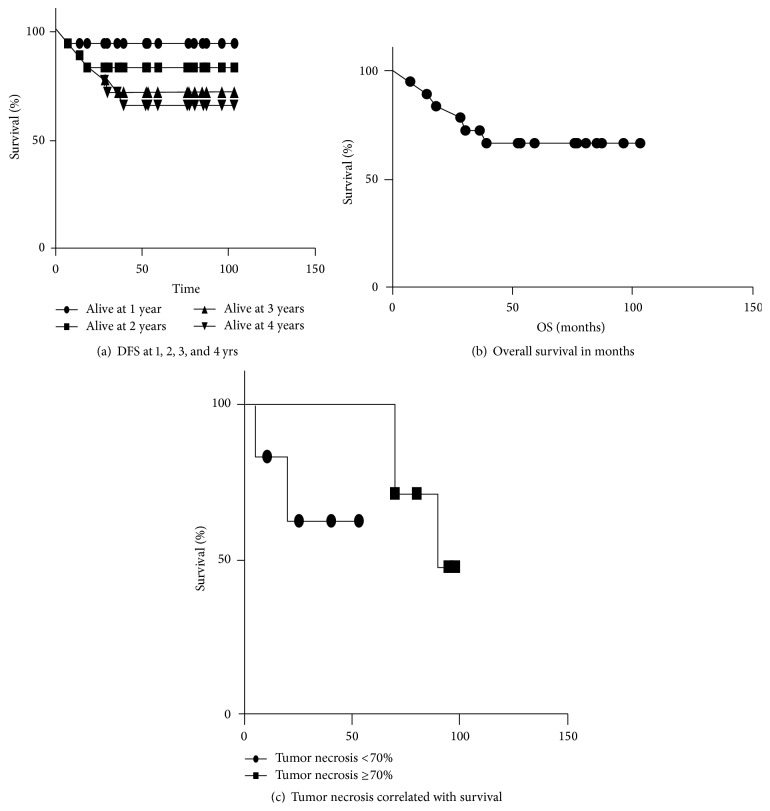
(a) Kaplan Meir curves showing the disease-free survival at 1, 2, 3, and 4 years (94%, 83%, 72%, and 66%, resp.) of patients who received neoadjuvant intratumoral injection of dendritic cells and radiation in high-grade soft tissue sarcomas over 5 cm. (b) Kaplan Meir curve showing overall survival of patients in months on patients who received neoadjuvant intratumoral injection of dendritic cells and radiation in high-grade soft tissue sarcomas over 5 cm. (c) Kaplan Meir curves showing tumor necrosis less than 70% or ≥70% in patients who received intratumoral dendritic cell injections followed by radiation and then surgery with resection of tumors to determine the percentage of necrosis after surgery *p* = 0.76.

Figure 1(a) displays DFS of study patients. A total of 7 patients have had disease progression and the DFS estimate at two years is 83% and at four years is 67%.  Figure 1(b) displays OS of study patients. A total of 6 patients have died, and the median time to progression was 142 days. We observed clinical responses in high-grade soft tissue sarcomas over 5 cm in size to be 67% from this study that is superior to the previously published outcomes of 45–55% [1, 3, 7, 8].

Of the eight patients that show disease-free survival between six and eight years, five had positive immune responses either to tumor cell lysate and/or survivin. Comparing survival in immune responders versus none responders using a two-tailed unpaired *t*-test showed that there was no difference between the two groups (*p* = 0.16, CI = −27.68 to 55.18).

### 3.4. Accumulation of Myeloid-Derived Suppressor Cells and Regulatory T Lymphocytes

The primary endpoint of the study was to examine the immunologic T-cell response to stimulation with specific tumor associated antigens (tumor cell lysate and survivin) as published before [12].

### 3.5. Correlation of Immunological with Clinical Response

**Figure 2:**
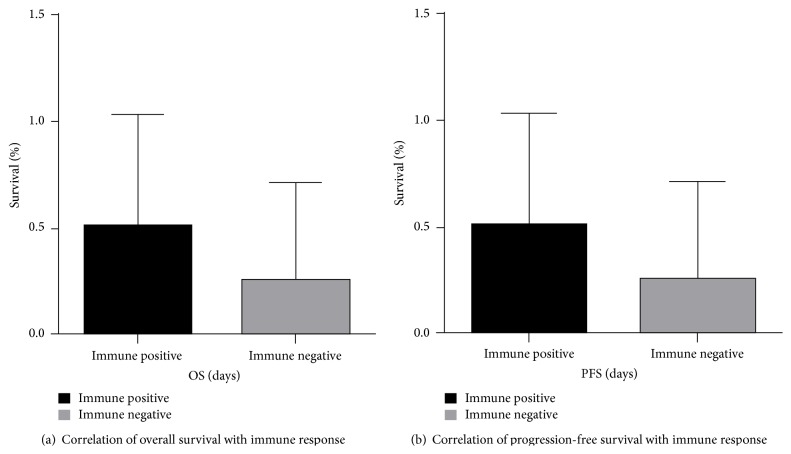
The eighteen patients who received neoadjuvant radiation and DC vaccine were divided into two groups based on their immune response (positive and negative) and then correlated with the overall survival in days and progression free survival.

Ten out of 18 individuals (56%) demonstrated evidence of an immune response to either tumor cell lysates (TCL) or survivin, at least at one point after the start of the treatment. Seven patients showed a response to only TCL, two patients to only survivin, and four to both TCL and survivin (Table 3). Tumor necrosis did not correlate with survival using log-rank test (Mantel-Cox) *p* = 0.08 (Table 3, Figures 2(a) and 2(b)). We found that there were some patients whose immune responses correlated with prolonged survival (Table 3). Due to their small numbers, this was not statistically significant and warrants further evaluation through prospective randomized controlled clinical trials.

### 3.6. Case Report

**Figure 3:**
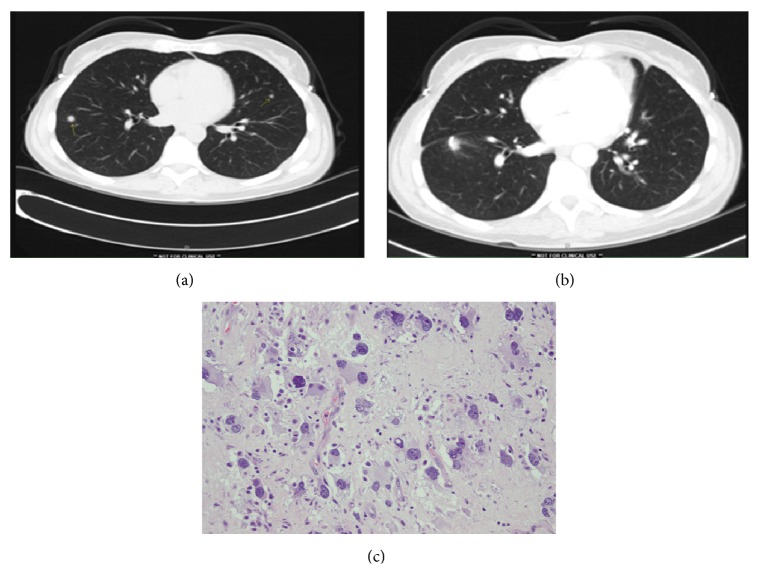
Pre- and postsurgery CAT scan of a 46-year-old lady with history of dedifferentiated liposarcoma patient who completed the DC vaccine + RT treatment, underwent surgical resection, and approximately 10 months later presented with (a and b) pre- and post-CT scans showing the presence of the lung nodules (two) and then postsurgical imaging study after its resection. (c) Pathological analysis showed tissue necrosis, hyalinization, and infiltration of lymphocytes and is currently free of systemic or local recurrence over 8 years.

A 46-year-old lady, who received neoadjuvant radiation and DC vaccine, had minimal side effects from the vaccine and a positive immune response to tumor antigens and survivin. Subsequent CAT scans a year later showed lung nodules suspicious for metastatic disease. These were surgically resected showing fibrosis, hyalinization, and infiltration of reactive lymphocytes on histopathological analysis (Figures 3(a), 3(b), and 3(c)).

## 4. Discussion

From this neoadjuvant clinical trial, we find that the dendritic cell injections were well tolerated with minimal side effects. Durable clinical responses were seen in these preliminary trials when compared to standard treatments with previously published results [1, 3, 4, 7].

First of all, these exploratory trials were designed with the intention of piloting DC injections for safety and immune response assessment in STS. In this study 12 out of 18 patients (67%) were alive with no systemic recurrence at eight years, while 6 out of 18 (33%) progressed to metastatic disease to the lung in 3 years. The fact that significant clinical responses are seen in these patients led to this report, indicating that the DC vaccines warrant further clinical evaluation by prospective randomized clinical trials. The immune change in the tumor microenvironment correlating with such prolonged remissions is what we need to focus on with appropriate correlative research while designing clinical trials in the future.

In these studies, leukapheresis followed by* ex vivo* expansion with standardized protocols was utilized to obtain expanded autologous DCs from mononuclear cell fraction progenitors that are HLA-matched (autologous) and competent to take up tumor-derived apoptotic bodies and present tumor antigens to lymphocytes. Bridging the gap in the DC maturational pathway caused by tumors defeats the tumor-induced immune tolerance seen in cancer patients. In our murine model, there was a direct relationship between acquisition of detectable tumor-specific cytotoxic T lymphocyte activity and clinical cure.

Dendritic cell (DC) therapy has not yet been validated, as there is no standardized method for its preparation [20, 21], timing of administration, and lineages of dendritic cells used (mononuclear or plasmacytoid) [24]. Several vaccine-based trials have been reported but have limited efficacy.

Traditionally radiation therapy has been viewed as immunosuppressive, but its immune functions can be complex. There is published data that radiation doses below 20 Gy do not affect the antigen-presenting function of DCs* in vitro* [15]. Thus, it is unlikely that the radiation at 2 Gy per fraction would have any significant effect on DC functions, even if the cells were very slow to migrate. The exact dose and delivery method of radiation with DC vaccine are still unknown and currently under evaluation.

This exploratory analysis is one of the first attempts at initiating DC vaccine therapy for soft tissue sarcoma. The primary objective of establishing immunological responses following DC therapy combined with radiation in soft tissue sarcoma was confirmed along with significant clinical responses in these patients, which warrants comparative trials with controlled treatment groups that will help define therapeutic efficacy.

## Figures and Tables

**Table 1:** Demographics and clinical characteristics of patients enrolled on combined treatment of neoadjuvant radiation and dendritic cell injections.

Demographics	Phase I
*Age*	
<61	10
>61	8
*Gender*	
Female	4
Male	14
*Primary site*	
Upper extremity	7
Lower extremity	11
*Histology*	
Epithelioid sarcomas	2
Sarcomas with myxoid features	2
Pleomorphic sarcomas	10
Others	4
*Stage*	
T2N0M0 Stage IIB	1
T2NxM0 G3 Stage III	17

**Table 2:** Common adverse events of Phase I trial combining neoadjuvant radiation and dendritic cell injections subdivided by grades 1–5 and total number of events during the course of the treatment.

	Grade 1	Grade 2	Grade 3	Grade 4	Grade 5	Total
Pain	33	22	5	0	0	61
Rash	16	0	0	0	0	16
Erythema	19	0	0	0	0	19
Hyperglycemia	16	5	0	0	0	22
Limb edema	16	5	0	0	0	22
Hyper pigmentation	5	0	0	0	0	5
Injection site reactions	11	5	0	0	0	16
Pruritus	5	0	0	0	0	5
Fatigue	66	33	0	0	0	99
Back pain	5	11	0	0	0	16
Nausea	38	11	5	0	0	54
Bruising	5	0	0	0	0	5
Constipation	44	11	0	0	0	55
Allergic reaction/hypersensitivity	5	0	0	0	0	5

**Table 3:** Histological type, immune responses to tumor cell lysates, and proliferation and immune response to survivin, percentage of tumor necrosis, and survival in months data of 18 patients recieving neoadjuvant radiation and dendritic cell vaccine treatment.

ID	Histology	Age	Gender	Location	Survival in months	Tumor necrosis in %	Progression to lung metastases in days	Immune response to TCL proliferation	Immune response to TCL (IFN/Elispot)	Immune response to survivin
1	Myxoid liposarcoma	41	M	Groin	103	NA		Positive	Positive	Negative

2	Synovial sarcoma	53	M	Right shoulder	96	NA	951	Negative	Positive	NA

3	Dedifferentiated liposarcoma	46	F	Right thigh	103	70%		Negative	Negative	Positive

4	Spindle/ pleomorphic	69	F	Left knee	59	98%		NA	NA	NA

5	Undifferentiated pleomorphic sarcoma	64	F	Left pelvis	80	80%		Negative	Negative	Negative

6	Sarcoma with myxoid and chondroid features	73	F	Lumbar spine	53	53%		Negative	Negative	Negative

7	Pleomorphic sarcoma	79	M	Left arm	76	25%		Positive	Negative	Negative

8	Undifferentiated pleomorphic sarcoma	71	M	Left thigh	14	70%	142	Negative	Negative	Positive

9	Myxoid liposarcoma	55	M		52	NA		Positive	Positive	Negative

10	Fibrosarcoma	73	M	Back	85	NA		Positive	Positive	Negative

11	Spindle cell sarcoma	81	M	Right flank	77	10%		Negative	Negative	NA

12	Synovial sarcoma	25	M	Left thigh	28	NA	146	Positive	Negative	NA

13	Fibrosarcoma	52	M	Right thigh	30	20%	132	Positive	Negative	NA

14	Undifferentiated pleomorphic sarcoma	58	M	Left thigh	7	90%	48	NA	NA	NA

15	Synovial sarcoma	34	M	Left knee	39	70%	947	NA	NA	NA

16	Undifferentiated pleomorphic sarcoma	60	M	Right leg	87	40%		Negative	Negative	NA

17	Spindle cell sarcoma	81	M	Right flank	36	95%		NA	NA	NA

18	Undifferentiated pleomorphic sarcoma	50	M	Right paraspinal	18	5%	102	Positive	Negative	NA
